# Supplementary material for: Reaching culturally acceptable and adequate diets at the lowest cost increment according to income level in Brazilian households
Source: PLoS One. 2020 Mar 11;15(3):e0229439. doi: 10.1371/journal.pone.0229439 (PMC7065914; doi:10.1371/journal.pone.0229439)
Supplement: Attachment — Submitted filename: paper_submited_with_comments.docx [file pone.0229439.s002.docx]

**Title page**

**Reaching culturally acceptable and adequate diets at lowest cost increment according to income level in Brazilian households**

**Short title:** The lowest cost of a nutritionally adequate diet for the Brazilian population

Eliseu Verly-Jr ^1^, Nicole Darmon ^2^, Rosely Sichieri ^1^, Flavia Mori Sarti ^4^

^1^ Department of Epidemiology, Institute of Social Medicine, Rio de Janeiro State University, Rio de Janeiro, Brazil.

^2^ MOISA, INRA, CIHEAM-IAMM, CIRAD, Montpellier SupAgro, Université de Montpellier, Montpellier, France.

^4^ Center for Research in Complex Systems Modeling, School of Arts, Sciences and Humanities, University of São Paulo, São Paulo, Brazil.

Corresponding author: Eliseu Verly-Jr eliseujunior@uerj.br

**Abstract**

**Objective**: To identify food choices allowing the fulfillment of a nutritionally adequate diets resembling actual food patterns at the lowest cost achievable for the Brazilian population, stratified by income level. **Methods**: Food consumption and prices were obtained from the Household Budget Survey (n=55,970 households) and National Dietary Survey (n=32,749 individuals). The sample was stratified into capitals of the states, rural areas, metropolitan areas, and rest of the state, and further by income levels according to the minimum wage (totaling 350 geographic-income strata, or GIS). Linear programming models were performed for each GIS in order to find the lowest cost of diets that meet a set of nutritional constraints. In order to find realistic diets, constraints referring to preferences were introduced in the models allowing optimized food quantities to depart progressively from the current intake for each food item and food group. The impact of meeting each target nutrient was assessed by performing models removing each nutrient at the time. **Results**: The observed and optimized diet costs were US$1.86 and US$2.58. The highest cost increment and the greatest food shifts were observed in the lowest income level. The nutrient adequacy was reached by mainly increasing fruits and vegetables, beans, fish and seafoods, non-fat milk, dairy, nuts, and eggs; and reducing red meat, chicken, margarine and butter, rice, processed meat, sugar-sweetened beverages (SSB), and sweets. As the departure from the current intakes increases, the cost difference tends to values lower than zero in the highest income, but not less than US$0.40 in the lowest income level. Calcium was the most expensive nutrient to meet adequacy. **Conclusion**: Nutritionally adequate diets are possible but costlier than the observed.

**Introduction**

Nutrient intake recommendations, such as those from Dietary Reference Intakes, are used worldwide for several purposes in the nutrition field, especially for dietary planning and assessment [1], food guide development [2], and fortification and nutrition-related policies [3]. Studies from developed [4,5] and developing countries [6] have shown high prevalence of inadequate intakes of some nutrients.

Food prices are one of the most important determinants of food choices, and cost constraints are well-known barriers to the adoption of healthy food choices in populations with low-socioeconomic status (SES) [7]. Early observational studies of dietary intake in Australia [8], UK [9], and France [10], have found that healthier diets tend to be more expensive than less-healthy diets, a finding later confirmed in the US [11] and worldwide [7,12]. In fact, when the budget for food is very low, it is logical to select foods that are cheaper sources of calories [13], but those foods generally have high energy density and low micronutrient density [14], leading to unhealthy dietary intakes [13,15]. Thus, if improvements of dietary intakes induce extra-cost to the consumer, they are not likely to be adopted in the population, especially in low SES population, where a high percentage of the total income is already assigned to food purchase. For example, in Brazil for 21% of households the total income was up to two minimum wages; in these households, the percentage of the total income spend on food was 27.8%, while this value for the whole population was 16.1% [16]. On the other hand, food consumption changes toward a healthy diet might be hampered by food preferences, even in the context where cost is not an important constraint on food purchasing. For example, it is a consensus from the literature that richer people eat more fresh foods than poorer ones, however it does not ensure that they have, or are close to a healthy diet [17].

Linear programming is an analytic method for the optimization of variables, subject to constraints expressed as target values that should be met. It helps in the assessment the feasibility of complex problems involving multiple variables and constraints (such as cost and nutrient contents) and to find their optimal solution [18]. Results from studies with diet optimization may support decisions on food policies and nutritional guidelines after considering constraints of cost and acceptability, indicating what could be more effective and efficient in terms of diet changes, i.e., higher quality and acceptability and lower cost.

Diet optimization studies based on dietary data in France demonstrated that it is possible to decrease the cost of a nutritious diet at the expense of social acceptability [19]. Using linear programming, they found a range of cost for nutritionally adequate diets, but this resulted in greated change in the observed diets when the budget was restricted, that is, the reduction of the distance between current consumption and the optimized food plans led to higher monetary costs

While it is known from some studies in developed countries that nutritional adequacy can be reached with realistic modifications within the habitual food patterns of individuals at no or little cost increment [20], there is no evidence that it may apply to developing countries, especially for low-income subpopulations. In this study, we aimed at identifying the food choices allowing to fulfill a nutritionally adequate diet that most resemble the actual food pattern at the lowest cost achievable for the Brazilian population, stratified by income level.

**Methods**

**Surveys**

We used data from the National Dietary Survey (NDS) and the Household Budget Survey (HBS), both conducted in 2008 and 2009 by the Brazilian Institute for Geography and Statistics. NDS was simultaneously collected in a random subsample of ~25% of the HBS. A two-stage sampling process was adopted: in the first stage, census tracts were randomly selected; and, in the second stage, households were randomly selected within census tracts.

Census tracts (n=12,800) were grouped into 550 household strata with geographical and socioeconomic homogeneity, and the number of tracts in each stratum was proportional to the number of households in the stratum. The samples included 55,970 households (HBS) and 13,569 households (NDS). Household visits in each stratum were uniformly distributed throughout the 12-month period to encompass seasonal variations in both food intakes and prices. More information on the surveys and data collection can be found elsewhere [21].

**Unit of analysis**

Considering that Brazil is a large and heterogeneous country, food patterns and prices widely vary throughout its macro and micro regions. To accommodate this variation in the elaboration of the lowest-cost optimized diets, linear programming models can be estimated for each sampling stratum; in this case, the model inputs and the solution (i.e., the optimized diet) are stratum-specific.

The 550 household strata were collapsed into four higher geographic areas: capital of the state, metropolitan region, rest of the state, and rural area, and further stratified into income level according to the *per capita* income: ≤0.5 official minimum wage (MW), >0.5 and ≥1.5 MW, >1.5 and ≤3 MW, and > 3 MW (Minimum wage: BRL415.00 (Brazilian Reals) equivalent to US$179.65 in January 2009). The procedure was performed in each of the 26 Brazilian States and one Federal District, totaling 350 aggregated strata (named geographic-income strata, or GIS; noting that in the sampling process, not all states were stratified into these four geographic areas).

This rearrangement was adopted to improve the precision of the estimates by increasing the number of households by each unit of analysis. Due to the long period of data collection, family income was adjusted to the same reference date (January 31^th^ 2009) using official inflation rates (National Consumers’ Prices Index) to allow comparability between households visited several months apart.

**Model inputs**

**Dietary intakes**

Dietary intake based on the NDS was collected from two non-consecutive food records filled by 32,746 individuals ≥10 years old (pregnant and breastfeeding women excluded; n=1,254). Participants reported the consumption of 305 foods, mostly clustered into 101 food items (e.g., different types of cakes were categorized into ‘cakes’), further classified into 26 food groups.

**Food prices**

Food prices were extracted from HBS database, where each household registered the amount and price of each food product purchased, further converted into prices per 100g of edible portion. Prices of foods collapsed into a single food product (e.g., different types of orange into ‘orange’) was estimated as the mean price of the foods weighted by the frequency of reporting in the budget survey.

Prices for each food product purchased were matched to the corresponding food item declared as consumed in the dietary intake survey according to the GIS, thus, the price variation over the GIS was preserved. Considering the variation in food prices throughout the period of collection, all prices were deflated to the same reference date (January 31^th^ 2009) using official inflation rates.

**Mean observed food intakes and mean observed cost**

Mean food intakes were calculated for each GIS considering sampling weights. Overall mean food intakes, i.e., the mean intakes over all 350 GIS, is referred as ‘mean observed diet’. Likewise, overall mean diet cost (i.e., the mean diet cost from all the 350 GIS) is referred as ‘mean observed cost’.

We excluded non-food nutrient and energy sources from the food items list, i.e., coffee and tea (without sugar), and alcoholic beverages, resulting in a list varying from 8 to 82 food items, according to the GIS. The set of food items declared as consumed in each GIS is designed as the GIS-specific food repertoire.

The recently launched Brazilian Food Composition database (TBCA-USP) was used to obtain nutrient content in both observed and optimized diets. Nutrient composition of foods clustered into food items (e.g. different types of rice into ‘rice’) was estimated as the mean composition of the foods weighted by the frequency of reporting in the dietary survey. The diets consumed in each GIS, namely the GIS-specific food repertoire and the GIS-specific distribution of food groups intakes, were used as starting point for the optimized diets derived from in the linear programming models.

**Linear programming models**

A linear programming model is defined by an objective function which is optimized (i.e., minimized or maximized), depending on decision variables restricted by various constraints [19]. In the present study, decision variables were the quantities (in grams) of foods from the GIS-specific food repertoire. Food items not reported to be consumed by any individual in a given GIS were allowed to be introduced as decision variable when they were reported by individuals from another GIS within the same state. We develop linear programming models to obtain the lowest cost of nutritionally adequate diets for each GIS, with the objective function described as follow:

$$min y= \sum_{i=1}^{i=n} (Q_{f,g}^{opt}\cdot{price}_{f,g}) [1]$$

Where *y* represents the objective function to be minimized, *i* is the food item reported in the GIS *g* from a set of *n* food items consumed, $Q_{f,g}^{opt}$ and the ${price}_{f,g}$ are, respectively, the optimized quantity and the price of the food item *f* in *g*.

**Models constraints**

The realism of the optimized diet, defined as how much it resembles the current observed diet, was reached by imposing two set of acceptability constraints as described below:

Acceptability food item constraints: These are boundaries in which optimized quantities of food items may deviate from the observed mean intakes in order to avoid optimized diets being culturally or socially unacceptable. Acceptability constraints may include lower and upper values, that is, the lowest and highest amount for a given food item allowed in the models. Some studies using linear programing have applied acceptability constraints derived from the population intake distribution, such as 10^th^, 20^th^, 80^th^ or 90^th^ percentile of intake [19]. In Brazil, a previous study showed that meeting all nutrients is mathematically impossible considering boundaries for each food item derived from the population intake distribution [22], meaning that, in order to fulfil all nutritional constraints, more flexible boundaries should be allowed. In order to find the lowest deviation from the observed diets consistent with a feasible solution, and to assess the relationship between cost and acceptability, the boundaries were introduced in the models allowing optimized food quantities to vary progressively from the observed mean intake for each food item. It was done by performing 300 models for each GIS, imposing upper boundaries ***ub*** that consist in $[m_{f,g}+d]$, where $\boldsymbol{m}_{\boldsymbol{f,g}}$ is the mean quantity of the food item ***f*** observed in a given GIS ***g****,* and ***d*=(1,2,3, …, 300)** is the allowed deviation from the observed amount reported of each food item ***f*** (hereafter referred just as ‘deviation ***d***’). The constraint ***ub*** for the food item ***f*** was, however, censored to its GIS-specific current mean portion size, defined here as the mean consumption in a consumption-day. That is, the food item ***f*** content allowed in the optimized diet should not be higher than what people eat, in average, in the day when the food item is eaten. The lower boundaries ***lb*** were obtained from the equation ($[1/(ub/m_{f,g})]*m_{f,g})$. The rationale behind this equation is that the increase and decrease allowed for a given food item is proportional to deviation ***d*** (e.g., if a given food item is allowed to double the amount, it will consequently be allowed to be reduced by half). Furthermore, it prevents the total removal of a given food item from the GIS-specific food repertoire because the ***lb*** never gets to zero.

Acceptability food groups constraints: Additional constraints were imposed on quantities from each food group. In each GIS model, food group quantities were not allowed to be higher than a defined boundary ***p*** to avoid unrealistic diets. These boundaries (***p***) were derived from the percentiles distribution of the amounts eaten of each food group in a consumption day (i.e., considering only consumers), stratified by state. Thus, the boundaries assigned to each GIS were established according to its corresponding state. The ***p*** constraints introduced in the models referred to the 50^th^, 55^th^, 60^th^, 65^th^, 70^th^, 75^th^, 80^th^, 85^th^, and 90^th^ percentiles. Thus, it was performed 2,700 models for each GIS (300­*_d_* * 9*_p_*), totaling 945,000 (2,700 * 350*_g_*) models. In case of model unfeasibility for a given GIS, we performed the analysis for the GIS not censoring the ***ub*** to the mean portion size.

Nutritional constraints: Constraints for calcium, magnesium, iron, phosphorus, copper, zinc, vitamins A, B1, B2, B6, B12, C, niacin, and folate were introduced in the models. Values were derived from the Estimated Average Requirement (EAR) [1]; as they are age-sex specific, overall constraint for each nutrient corresponded to mean values of requirements (i.e., mean of age-sex EAR values) weighted by frequency of age-sex group in the population. Nutrients without EAR were not constrained in the models. Total energy content was constrained to be equal to the mean estimated energy requirement (EER) [24] calculated using age, sex, and anthropometric information specific for each GIS (mean EER over the 350 GIS = 2,113 kcal). Due to absence of information on accuracy of estimates for added salt in food preparations, the ratio sodium/kcal in optimized diet was constrained to be equal or lower than the ratio in the observed diet obtained for each GIS. Saturated and *trans* fats, and added sugar constraints were based on the WHO Report [24]. The whole set of nutritional constraints introduced in all of the models, including macronutrients, fats and sugar, as well as the reference for each recommendation is presented in **Table 1**.

**Table 1 – Nutritional constraints imposed in the models.**

| **Component** | **Constraint** |
| --- | --- |
| Energy (kcal) | = EER^a^ |
| Proteins | 10% - 35% kcal^a^ |
| Carbohydrates | 45% - 65% kcal^a^ |
| Total fats | 20% - 35% kcal^a^ |
| Saturated fat | <10% kcal^b^ |
| Trans fat | < 1% kcal^b^ |
| Added sugar | <10% kcal^b^ |
| Total fiber | ≥ 30g^c^ |
| Sodium/kcal | ≤ observed ratio^d^ |
| Calcium | ≥ 868mg^c^ |
| Magnesium | ≥ 303mg^c^ |
| Phosphorus | ≥ 649mg^c^ |
| Iron | ≥ 6.8mg^c^ |
| Copper | ≥ 0.7mg^c^ |
| Zinc | ≥ 8mg^c^ |
| Vitamin A | ≥ 560mg^c,e^ |
| Vitamin B1 | ≥ 0.9mg^c^ |
| Vitamin B2 | ≥ 1mg^c^ |
| Vitamin B6 | ≥ 1.1mg^c^ |
| Vitamin B12 | ≥ 2mcg^c^ |
| Vitamin C | ≥ 66.1mg^c^ |
| Niacin | ≥ 11.5mg^c^ |
| Folate | ≥ 322mcg^c,f^ |

^a^ Estimated Energy Requirement [23].

^b^ World Health Organization [24].

^c^ Derived from the Estimated Average Requirement [1].

^d^ Ratio sodium/kcal observed in each GIS.

^e^ Retinol Activity Equivalents.

^f^ Dietary Folate Equivalents.

**Influence of the nutrient targets on cost and tolerability**

In order to assess the impact of meeting adequacy for each nutrient on the cost and the acceptability, the models were performed removing the constraint for one nutrient at a time. For example, say a model was estimated keeping all nutritional constraints but removing calcium constraint, then, performed again keeping all nutritional constraints (including calcium) but removing the zinc constraint, and so forth for all nutrients.

Linear programming models were estimated using the Optmodel Procedure from software SAS OnDemand.

**Descriptive analyses**

The impact of meeting nutritional adequacy on cost and food group changes was assessed by comparing the cost differences across the solutions for the range of deviation ***d*** introduced progressively in the models. In this analysis, each GIS could reach the nutritional adequacy at different deviation, resulting in different cost and food changes. Thus, for those 300*_d_* * 9*_p_* costs-minimization models for each GIS, we ascending ranked the solutions by the deviation ***d*** and percentile ***p***, so that we have the solution with the lowest deviation for each GIS, and then the second lowest deviation for each GIS, and the third, and so on, till the ***k*** solutions.

In fact, for a given GIS and deviation ***d***, more than one solution with different ***p*** could be reached; when it happened, we selected the one with the lowest ***p***. The mean cost and deviation over the 350 GIS were computed for each ***k*** solution. The first ***k*** solution was that in which each GIS reached the nutritional adequacy at the lowest deviation, then it was considered the best solution, that is, the one with the lowest cost at the lowest deviation from the observed diet.

The mean optimized cost, mean cost difference, mean food changes, and mean deviation ***d*** were presented for the whole sample and stratified by income levels. The cost difference (*CD*) was calculated according to the following equation:

$${CD}_{g}=\left( \sum_{i=1}^{i=n} {price}_{f,g}\cdot Q_{f,g}^{opt} \right)-\left( \sum_{i=1}^{i=n} {price}_{f,g}\cdot Q_{f,g}^{obs} \right) [2]$$

where:${price}_{f,g}$, $Q_{f,g}^{opt}$, and $Q_{f,g}^{obs}$ are respectively the GIS-specific price, the optimized quantity, and the observed quantity of the food item *f* in the GIS *g.*

The relationship between cost and deviation was assessed by plotting the cost difference with the mean deviation across the ***k*** solutions, also stratified by income levels. Furthermore, we presented the results across the five first ranked ***k***-solution (optimized diets that most resemble the observed diet) in order to assess the relationship between cost and deviation in the changes in food group quantities compared with the observed diet. Finally, we used the same procedure to select the best solution for the models assessing the impact of each nutritional constraint on the cost and deviation.

**Ethics**

The protocol of this research was approved by the Ethics Committee of the Instituto de Medicina Social of the Universidade do Estado do Rio de Janeiro (CAAE 0011.0.259.000-11).

**Results**

Among the 2,700 models ran for each of the 350 GIS, most of the feasible solutions were achievable for the deviation ***d*** (allowed deviation for each individual food amount in *g*) between 20g and 80g, and ***p*** (maximum allowed amount for each food group in *g*) ≤60^th^ GIS-specific percentile.

**Selected best solutions**

For the selected best solutions, the sum of all the positive changes (increases in food quantities after optimization) was, in average over all GIS, 475g, and the sum of all negative changes (decreases in food quantities) was, in average over all GIS, 183g; the overall increase in the food quantities was, in average over all GIS, 292g.

The food repertoires after optimization varied from 24 to 84 items over the 350 GIS. **Table 2** shows the mean cost of the observed and best optimized diets, the mean cost difference, and the mean deviation ***d*** over all GIS. Results are presented for Brazil and stratified by monthly per capita income in minimum wage.

**Table 2** **– Mean (standard error) observed diet cost, in US$, mean cost of optimized diets^a^, mean cost difference and mean deviation *d* (in g/d) from the observed diets, according to income level (n=350 GIS^b^).**

|  | Observed diet cost | Optimized diet cost | Cost difference^d^ | ***d***^e^ |
| --- | --- | --- | --- | --- |
|  |  |  |  |  |
| <0.5MW^c^ | 1.49 (0.05) | 2.28 (0.11) | 0.78 (0.11) | 78.24 (4.94) |
| 0.5-1.5MW | 1.72 (0.02) | 2.41 (0.07) | 0.69 (0.08) | 57.64 (4.30) |
| 1.5-3MW | 1.97 (0.03) | 2.67 (0.06) | 0.70 (0.06) | 50.30 (3.25) |
| >3MW | 2.25 (0.04) | 3.00 (0.07) | 0.75 (0.10) | 52.36 (3.53) |
|  |  |  |  |  |
| Brazil | 1.86 (0.03) | 2.58 (0.05) | 0.72 (0.04) | 57.72 (2.36) |

^a^ Lowest cost at the lowest deviation from the observed diet.

^b^ Geographic-income strata.

^c^ Minimum wage.

^d^ (mean optimized diet cost – mean observed diet cost).

^e^ Mean of ***d*** (acceptability food constraint introduced in the models).

The mean observed diet cost (per person/day) in the population was US$1.86 (BRL4.29), ranging from US$1.49 (BRL3.44) (lowest income) to US$2.25 (BRL5.19) (highest income). The mean cost of the optimized diet was US$2.58 (BRL5.96), ranging from US$2.28 (BRL5.26) in the lowest income to US$3.00 (BRL6.93) in the highest income. The cost increment for the nutritionally adequate diet was higher for the lowest income compared with the other income levels, although it was similar to that observed in the highest income.

However, the deviation from the observed diet needed to reach adequacy at this cost difference was higher in the lowest income level, compared with the other levels. The main changes to reach adequacy included increases in fruits and vegetables, beans, fish and seafoods, non-fat milk, dairy, nuts, and eggs, while red meat, chicken, margarine and butter, rice, processed meat, SSB, and sweets were the food groups with the highest reduction (**Table 3**).

**Table 3** **– Mean (standard error) food contents in the observed and optimized diets^a^ (n=350 GIS^b^).**

| Foods / Food groups | Observed diet | Optimized diet |
| --- | --- | --- |
| Beans | 182.2 (6.5) | 226.9 (5.3) |
| Breads | 63.4 (1.5) | 49.9 (2) |
| Cake | 13.2 (0.5) | 30.3 (2.4) |
| Cheese | 8.9 (0.7) | 26.2 (0.6) |
| Chicken | 38.5 (1) | 17.8 (0.7) |
| Cookies | 16 (0.7) | 10.9 (1) |
| Eggs | 10.7 (0.5) | 38 (0.7) |
| Fish / seafoods | 25.3 (2.5) | 60.4 (4.5) |
| Fruits | 128.9 (4.7) | 194.5 (2.1) |
| Leafy vegetables | 12.1 (0.7) | 23 (0.5) |
| Manioc flour | 7.7 (1.1) | 25.9 (1.2) |
| Margarine / Butter | 9.5 (0.4) | 2.9 (0.2) |
| Milk | 103.2 (2.4) | 98.4 (2.7) |
| Non-fat milk | 5.2 (0.6) | 56 (1.7) |
| Nuts | 0.2 (0) | 16.5 (0.4) |
| Olive Oil | 0 (0) | 0.2 (0.1) |
| Other vegetables | 28.7 (1.4) | 49.3 (0.7) |
| Pasta | 59.3 (1.9) | 81.5 (3.9) |
| Processed meats | 9.1 (0.4) | 2.2 (0.3) |
| Red meat | 79.5 (1.6) | 40.2 (1.4) |
| Rice | 154.5 (4) | 119 (4.1) |
| Snacks | 19.2 (1.1) | 44.3 (2.4) |
| SSB | 138.3 (5.3) | 103.4 (5.2) |
| Sweets | 21.9 (0.9) | 9 (0.6) |
| Tuber | 26.2 (1) | 66.7 (1.2) |
| Whole cereals | 7.5 (0.9) | 20.9 (2.3) |
| Yogurt | 11.6 (1.1) | 62.1 (2) |

^a^ Lowest cost at the lowest deviation from the observed diet.

^b^ Geographic-income strata.

^c^ Sugar-sweetened beverages.

**Cost versus deviation**

**Figure 1** represents the cost differences (i.e. change in cost after diet optimization) in relation to the deviation ***d*** from the observed diet: each symbol represents the mean cost difference over the 350 GIS according to the distance ***d***; the solid curve is the predicted cost difference. The more realistic the diet (less deviation ***d***), the higher the cost increment. But this relationship was not linear: the cost increment was very high in the lowest deviation ***d***, then it decreased dramatically when increasing the deviation ***d***, and tended to stabilize near 0 or even negative values (small decreases in diet cost for very high deviation ***d***). The main changes in the food quantities for the he five solutions with the lowest deviance ***d*** in the figure are presented in the **Table 4**. In general, the lower the amount of cake, fish and seafoods, fruits, whole milk, and snacks across the solutions, the lower the diet cost.

**Figure 1 – Cost differences between optimized and observed diets, in relation to the deviation from observed diet^a^.** ^a^ Mean of deviation ***d*** (acceptability food constraint introduced in the models). Each symbol represents the mean cost difference over the 350 GIS according to the distance ***d***; the solid curve is the predicted cost difference.

**Table 4** **– Mean foods change^a^ and overall mean cost difference^a^ according to the five solutions that most resemble the observed diet^b^.**

| Foods / Food groups | 1^st^. | 2^nd^. | 3^rd^. | 4^th^. | 5^th^. |
| --- | --- | --- | --- | --- | --- |
| Beans | 43.3 | 43.9 | 40.9 | 34.6 | 39.2 |
| Breads | -13.4 | -12.5 | -7.8 | -9.2 | -2.7 |
| Cake | 16.0 | 12.4 | 8.1 | 13.0 | 5.0 |
| Cheese | 17.8 | 17.7 | 17.6 | 17.2 | 17.5 |
| Chicken | -20.6 | -21.3 | -21.9 | -22.0 | -22.7 |
| Cookies | -5.2 | -1.3 | 1.7 | 1.3 | 1.6 |
| Eggs | 27.5 | 26.6 | 25.5 | 24.8 | 24.2 |
| Fish / seafoods | 37.2 | 17.6 | 11.3 | 17.4 | 4.8 |
| Fruits | 63.1 | 60.3 | 57.5 | 53.2 | 54.5 |
| Leafy vegetables | 10.6 | 10.0 | 10.0 | 9.3 | 9.5 |
| Manioc flour | 18.8 | 18.5 | 18.5 | 18.0 | 18.3 |
| Margarine / Butter | -6.7 | -5.3 | -4.2 | -4.4 | -3.4 |
| Milk | -4.8 | -9.8 | -14.7 | -19.3 | -20.9 |
| Non-fat milk | 51.2 | 56.0 | 59.2 | 61.9 | 64.6 |
| Nuts | 16.2 | 15.9 | 15.6 | 15.3 | 15.5 |
| Olive Oil | 0.2 | 0.2 | 0.3 | 0.4 | 0.5 |
| Other vegetables | 20.1 | 19.6 | 18.8 | 18.5 | 18.2 |
| Pasta | 22.6 | 38.1 | 39.9 | 39.8 | 42.7 |
| Processed meats | -7.0 | -7.2 | -6.9 | -7.0 | -6.9 |
| Red meat | -40.2 | -41.9 | -43.2 | -44.4 | -45.1 |
| Rice | -35.2 | -33.9 | -31.0 | -31.3 | -22.0 |
| Snacks | 24.5 | 18.5 | 12.7 | 14.6 | 7.3 |
| SSB | -37.1 | -39.4 | -40.8 | -43.0 | -44.3 |
| Sweets | -13.0 | -13.2 | -13.8 | -13.7 | -13.2 |
| Tuber | 40.4 | 39.4 | 39.5 | 38.2 | 39.3 |
| Whole cereals | 15.2 | 21.5 | 25.6 | 20.9 | 23.3 |
| Yogurt | 51.1 | 54.2 | 57.4 | 59.6 | 60.1 |
|  |  |  |  |  |  |
| **Cost difference** | **0.72** | **0.46** | **0.40** | **0.43** | **0.27** |

^a^ mean of the difference (optimized – observed, in grams or US$) over the 350 GIS_k_.

^b^ lowest deviation ***d***.

^c^ Sugar-sweetened beverages.

The same pattern of non-linear relationship between the cost difference and deviation from the observed consumption was observed when stratified by income level. The cost difference in the higher income levels (> 1.5 MW) tends to values lower than zero with the increase of the deviance ***d***. In the 0.5-1 MW income level the cost increment tends to stabilize about US$ 0.20 and in the lowest income level the minimum cost increment is about US$ 0.40 (**Figure 2**).

**Figure 2 – Cost difference between optimized and observed diets, in relation to the deviation from observed diet^a^, for each income level (in MW^b^).** ^a^ Mean of deviation ***d*** (acceptability food constraint introduced in the models). ^b^ MW=minimum wage.

**Impact of each nutrient adequacy on the cost and deviation**

When performing the optimized models removing each nutritional constraint separately, all models presented the same or very similar results concerning both cost difference and deviation compared with the full model (model with all the nutritional constraints). The exception was for calcium, for which the cost difference was US$0.23 (BRL0.53) and the mean deviation ***d*** was 36.8; lower values compared with US$0.72 (BRL1.66) and 57.7 from the full model including the calcium constraint). The main impact on food quantities after relaxing calcium constraint, compared with the full model, were observed for beans (-19g), cheese (-23g), yogurt (-37g), fish (-39g), whole and non-fat milk (-47g), fruits and vegetables (-43g), chicken (+18g), red meat (+14g), breads (+20g), and rice (+22g) (data not shown).

**Discussion**

In this study, we aimed at finding the lowest cost for the optimized nutritionally adequate diets that most resemble the actual food patterns in Brazil. Results are not intended to be seen as strict quantities that should be eaten by everyone, but as an indication of the main changes to improve diet quality at lowest achievable costs. Besides, as this approach departs from actual diet cost and food habits, these results provide insights in establishing food guides and policies potentially more effective using optimization tools, especially in developing countries. For example, the results point to possibility of adopting incentives in production and/or distribution of certain key foods to reduce prices for promoting higher intake that would potentially result in higher diet quality with greater chance of adherence by the population.

Main implications of this study are as follow: First, the nutrient adequacy demands substantial changes in food choices. One of the main concerns when designing dietary recommendations and optimized diets is to ensure that it is locally acceptable. Unrealistic food quantities were prevented in the study by imposing acceptability boundaries ranging from more to less stringent constraints. However, it was possible to obtain solutions for most of the GIS at acceptability boundaries near 60^th^ percentile or less, and deviation from the observed intake between 20g and 80g. It means that each individual food was allowed to increase up to 80g (the amount allowed to decrease depends on the observed intake quantity), which led to a net overall increase, in average over the 350 GIS, of 292g/day.

Our results showed the most realistic diet within acceptable cost and preference to meet nutritional adequacy, although the optimization have resulted in considerable distance from the actual consumption for some items. For example, beans were increased by 40g/day to reach nutritional adequacy, although trends in Brazil have shown consistent decline in beans consumption throughout the last decades [25,26]. Fruits content in the optimized diets increased from 128g to 194g; however, it can be argued that the observed consumption was quite low; thus, the high increase compensates insufficient baseline consumption. Likewise, it might be challenging to increase the nuts consumption from 0.2g/day to 16.5g/day for the population. Considerable changes in some foods are necessary due to observation of a current diet that fails to meet most nutrient requirements. Moreover, the changes encompass more than only cost and food preferences, including aspects of convenience [27]. One of the main changes included substantial increase in fruits and vegetables consumption, which implies weekly visit to the market due to shorter shelf-life in comparison to other foods. In addition, cooking hard vegetables and beans requires more time on preparation and energy (electricity or gas). Commonly observed nutrient inadequacies were registered both in low- and high-income households, that is, even disregarding costs, changes to fulfill nutrient adequacy were substantial among different households in the Brazilian population [17].

Second, there was cost increments of approximately 38% in nutritionally adequate optimized diets, considering the diet with higher similarity to the current food consumption. It is worth noticing that under higher flexibility of diet composition, there were diets with lower costs. However, costs were associated with changes in diet composition in a non-linear pattern: small divergence of optimized diets in comparison to observed diets resulted in lower differences in costs Similar non-linear inverse relationship between cost and deviation from the observed diet was found in a study with French women: to meet nutritional constraints, the minimum departure from the observed diet was 495% at a cost of 4.99 euros, but to achieve nutritional constraints at the lowest possible cost (i.e., 3.18 euros), the deviation markedly increased to 2,870% [18]. In the present study, the minimum cost required to reach nutritional adequacy was higher than the cost of the observed diet, but the magnitude of this increase was greater for the lowest income groups. In this sense, irrespective of how much people in lower income groups tolerate substantial changes in the food choices, the adequacy probably will not be attained without increments in cost. Thus, nutritional counselling will not be sufficient unless poorer people increase food budget. In the lowest income level, the budget for food should increase in average by US$0.40 (BRL0.92) per capita per day, irrespective of the diet acceptability. On the other hand, in the highest income groups, adequacy can be attained at no cost or even with reduction of food budget. Comparing with studies applied to the French population, the cost increment in order to reach nutritional adequacy corresponded to +3.2% of the mean observed diet cost; and similarly to our results, the cost increment was higher in the lower income quintiles [20].

Third, we have shown that calcium recommendation is the costliest and the more demanding in terms of changes in food consumption. Deviation from the observed diet was substantially lower when the constraint for calcium was removed, in comparison to the solutions excluding constraints of other nutrients. Moreover, the impact of removing each of the nutrient constraints, with exception for calcium, was similar in terms of cost and acceptability in relation to the full nutrient-constrained models. Calcium is described as one of the nutrients with higher inadequacy in many populations [4,5]; in Brazil, the prevalence of inadequacy found in nationwide studies is more than 80% [6,17]. The difficulty to meet calcium requirement was already addressed in previous study in Brazil using linear programming [22]. On one hand, relaxing calcium constraint made the optimized diet cheaper and more realistic; however, on the other hand, the consequence of lack in dietary calcium is highly questionable. In a recent review on calcium intake and risk of bone fractures, the conclusions indicated no evidence that increasing calcium intake from dietary sources prevents fractures, and authors argued that recommendations of calcium intake required for bone health have been developed based on findings of early Ca-balance studies, which has never been shown to be associated with fracture risk [28]. In this line, the WHO report on nutrition and chronic disease states that a minimum of 400-500 mg of calcium intake would be sufficient to prevent osteoporosis in countries with a high fracture incidence [24], similar to what was obtained in the calcium-free model (564mg; data not shown).

Choices of inputs and constraints imposed in the optimization models need further considerations. The set of recommendations adopted (Dietary Reference Intakes) refers to populations of USA and Canada, taking into account factors as geography, anthropometry, and nutrient bioavailability from these populations. In this sense, we opted for excluding constraints for vitamin D and E in the models proposed, since vitamin D requirement assumes minimal sun exposition because of high imprecision in sunlight exposure due to skin pigmentation, genetics, latitude, use of sunscreens, and cultural differences in dressing habits, among other factors [29]. It is likely that tropical countries, such as Brazil, need less vitamin D from diet than countries from higher latitude, but to date, we cannot know how much the solar exposition by itself suffices the physiological vitamin D needs [30]. In addition, both DRI and WHO reports stated that there is insufficient information to define indicators for vitamin E adequacy, and they are mainly based on the mean intake observed in USA and other European countries [30,31].

The mean observed diet cost was derived from the actual food consumption reported in the food records. A validation study prior to the NDS collection estimated the underreporting energy intake about 30% comparing with energy expenditure from double-labelled water [32]. Thus, the distances between the observed and the optimized food quantities in the present study are probably overestimated. Furthermore, our results rely on the assumption that smaller differences between observed and optimized diets (in grams of food) ensure improved acceptability of the food changes.

Finally, it is important to point that a main limitation of the study refers to the fact that data collection was performed approximately ten years ago. However, it is also the most recent nationwide data on food consumption and prices to date. Additionally, this represents an innovative study that takes into account the populational strata in the diet modelling, accommodating actual food intakes and prices within nationally representative sets of households marked by geographic and socioeconomic homogeneity within cluster. To our knowledge, it is the unique study assessing food prices and consumption at the same household in the same period of collection. It is especially important in the context of large and heterogeneous country such as Brazil. For example, the mean daily fish intake varies from 6.8g in Southern to 95g in Northern Brazil.

In conclusion, the study shows that nutritionally adequate diets are possible, usually at higher costs in comparison to the observed dietary patterns in Brazil; and that cost increments presented lower impacts on household budgets in case of substantial changes in the current food patterns, being especially demanding in the lowest income population group.

**References**

1. Institute of Medicine. Dietary Reference Intakes: Applications in Dietary Assessment. National Academies Press. Washington DC. 2000.
2. European Food Safety Authority. Scientific Opinion of establishing Food-Based Dietary Guidelines. the Panel on Dietetic Products, Nutrition, and Allergies (NDA). The EFSA Journal 2010, 8, 1-42.
3. Vieira D, Steluti J, Verly-Jr E, Marchioni D, Fisberg R. Brazilians’ experiences with iron fortification: Evidence of effectiveness for reducing inadequate iron intakes with fortified flour policy. Public Health Nutr. 2017;20(2):363-370. doi:10.1017/S1368980016001981.
4. Roman Viñas B, Ribas Barba L, Ngo J, Gurinovic M, Novakovic R, Cavelaars A, et al. Projected Prevalence of Inadequate Nutrient Intakes in Europe. Ann Nutr Metab. 2011; 59:84-95. doi: 10.1159/000332762
5. Moshfegh A, Goldman J, Cleveland L. What we eat in America, NHANES 2001-2002: Usual nutrient intakes from food compared to Dietary Reference Intakes. US Department of Agriculture, Agricultural Research Service. Washington, DC, USA, 2005.
6. Fisberg RM, Marchioni DM, de Castro MA, Verly-Jr E, Araujo MC, Bezerra IN, et al. Inadequate nutrient intake among the Brazilian elderly: National Dietary Survey 2008–2009. Rev Saude Publica. 2013;47:222S–230S. doi: 10.1590/S0034-89102013000700008.
7. Darmon N, Drewnowski A. Contribution of food prices and diet cost to socioeconomic disparities in diet quality and health: a systematic review and analysis. Nutr Rev. 2015;73(10):643–660. doi: 10.1093/nutrit/nuv027.
8. McAllister M, Baghurst KI, Record S. Financial costs of healthful eating: a comparison of three different approaches. J Nutr Educ. 1994;26:131–139. doi: 10.1016/S0022-3182(12)80387-6
9. Cade J, Upmeier H, Calvert C, Greenwood D. Costs of a healthy diet: analysis from the UK Women’s Cohort Study. Public Health Nutr. 1999;2:505–512.
10. Darmon N, Briend A, Drewnowski A. Energy-dense diets are associated with lower diet costs: a community study of French adults. Public Health Nutr 2004;7:21–7. doi: 10.1079/PHN2003512.
11. Rehm CD, Monsivais P, Drewnowski A. The quality and monetary value of diets consumed by adults in the United States. Am J Clin Nutr. 2011;94:1333–1339. doi: 10.3945/ajcn.111.015560.
12. Rao M, Afshin A, Singh G, Mozzafarian D. Do healthier foods and diet patterns cost more than less healthy options? A systematic review and meta-analysis. BMJ Open. 2013;3:e004277. doi:10.1136/bmjopen-2013-004277.
13. Darmon N, Ferguson E, Briend A. Do economic constraints encourage the selection of energy dense diets? Appetite, 2003;41:315-322. doi: 10.1016/S0195-6663(03)00113-2.
14. Maillot M, Darmon N, Darmon M et al. (2007) Nutrient dense food groups have high energy costs: an econometric approach to nutrient profiling. J Nutr 137, 1815–1820. 10. doi: 10.1093/jn/137.7.1815.
15. Darmon N, Ferguson EL, Briend A. A cost constraint alone has adverse effects on food selection and nutrient density: an analysis of human diets by linear programming. J Nutr. 2002, Dec;132(12):3764-71.e. doi: 10.1093/jn/132.12.3764.
16. Instituto Brasileiro de Geografia e estatística. Despesas, rendimentos e condições de vida – Pesquisa de Orçamentos Familiares 2008-2009. Rio de Janeiro, 2010. Available from: <https://biblioteca.ibge.gov.br/visualizacao/livros/liv45130.pdf>

17. Araujo MC, Verly-Junior E, Junger W, Sichieri R. Independent associations of income and education with nutrient intakes in Brazilian adults: 2008–2009 National Dietary Survey. Public Health Nutr. 2014;17(12):2740-2752. doi:10.1017/S1368980013003005.

18. Gazan R, Brouzes C, Vieux F, Maillot M, LLuch A, Darmon N. A review on mathematical optimization: an effective tool to explore tomorrow’s sustainable diets. Adv Nutr. 2018;9(5): 602–616. doi: [10.1093/advances/nmy049](https://dx.doi.org/10.1093%2Fadvances%2Fnmy049).
19. Darmon N, Ferguson E, Briend A. Impact of a cost constraint on nutritionally adequate food choices for French women: an analysis by linear programming. J Nutr Educ Behav, 2006; 38:82-90. doi: 10.1016/j.jneb.2005.11.028.
20. Maillot M, Vieux F, Delaere F, Lluch A, Darmon N. Dietary changes need to reach nutritional adequacy without increasing diet cost according to income: An analysis among French adults. Plos One. 2017;12(13):e0174679.
21. Oliveira DCRS, de Moura Souza A, Levy RB, Sichieri R, Verly-Jr E. Comparison between household food purchase and individual food consumption in Brazil. Public Health Nutrition. 2018 ahead of print 1–7. Doi:10.1017/S1368980018002987.
22. Santos Q, Sichieri R, Darmon N, Maillot M, Verly-Jr E. Food choices to meet nutrient recommendations for the adult Brazilian population based on the linear programming approach. Public Health Nutr. 2018;18:1-8. Doi:10.1017/S1368980017003883.
23. Institute of Medicine. Dietary Reference Intakes for Energy, Carbohydrate, Fiber, Fat, Fatty Acids, Cholesterol, Protein, and Amino Acids. National Academies Press. Washington DC. 2005.
24. Joint WHO/FAO Expert Consultation on Diet, Nutrition and the Prevention of Chronic Diseases. Geneva, Switzerland, World Health Organization, 2002.
25. Levy-Costa RB, Sichieri R, Pontes NS, Monteiro CA. Household food availability in Brazil: distribution and trends (1974-2003). Rev Saúde Pública 2005;39(4):530-540.
26. Levy RB, Claro RM, Mondini L, Sichieri R, Monteiro CA. Regional and socioeconomic distribution of household food availability in Brazil, in 2008-2009. Rev Saúde Pública 2012;46(1):6-15. Doi: 10.1590/S0034-89102011005000088.
27. Machado PP, Claro RM, Canella DS, Sarti FM, Levy RB. Price and convenience: The influence of supermarkets on consumption of ultra-processed foods and beverages in Brazil. Appetite. 2017;116:381-388. doi: 10.1016/j.appet.2017.05.027
28. Bolland MJ, Leung W, Tai V, Bastin S, Gamble GD, Grey A, et al. Calcium intake and risk of fracture: systematic review. BMJ 2015;351:h4580. Doi: 10.1136/bmj.h4580.
29. Institute of Medicine. Dietary Reference Intakes for Calcium and Vitamin D. National Academies Press. Washington DC. 2011.
30. Joint FAO/WHO Expert Consultation. Vitamin and mineral requirements in human nutrition. Geneva, Switzerland, World Health Organization, 2004.
31. Institute of Medicine. Dietary Reference Intakes for Vitamin C, Vitamin E, Selenium, and Carotenoids. National Academies Press. Washington DC. 2000.
32. Lopes TS, Luiz RR, Hoffman DJ, Ferrioli E, Pfrimer K, Moura AS, et al. Misreport of energy intake assessed with food records and 24-h recalls compared with total energy expenditure estimated with DLW. Eur J Clin Nutr. 2016;70:1259–1264. doi: 10.1038/ejcn.2016.85.
